# Genetic and virulence factors behind the success of high-risk *Pseudomonas aeruginosa* clones: insights from comparative genomics and an experimental infection model

**DOI:** 10.3389/fmicb.2025.1674635

**Published:** 2025-10-31

**Authors:** Romário Oliveira de Sales, Laura Leaden, Dayblegschwel Santos Martins, Paula Koga, Alexandra do Rosario Toniolo, Fernando Gatti de Menezes, Marcelo Alves Mori, Marines D. V. Martino, Patricia Severino

**Affiliations:** ^1^Albert Einstein Research and Education Institute, Hospital Israelita Albert Einstein, São Paulo, Brazil; ^2^Laboratório Clínico, Hospital Israelita Albert Einstein, São Paulo, Brazil; ^3^Serviço de Controle de Infecção Hospitalar, Hospital Israelita Albert Einstein, São Paulo, Brazil; ^4^Institute of Biology, Universidade Estadual de Campinas, Campinas, Brazil

**Keywords:** *Pseudomonas aeruginosa*, high-risk clones, antimicrobial resistance, virulence factors, comparative genomics, *Caenorhabditis elegans*, genome-wide association study (GWAS)

## Abstract

**Introduction:**

*Pseudomonas aeruginosa* causes severe healthcare-associated infections. High-risk clones are defined by global dissemination and multidrug resistance, yet virulence is heterogeneous. We sought to map accessory-genome determinants associated with high-risk clones by integrating whole-genome sequencing (WGS) with a *Caenorhabditis elegans* infection model.

**Methods:**

We analyzed 84 clinical isolates plus publicly available genomes using WGS, phylogenomics, and resistome/virulome profiling. Virulence was measured by *C. elegans* slow-killing (SK). A GWAS of accessory-genome subelements (AGEs) identified *loci* with high- (HVA) or low-virulence association (LVA). Coding sequences were annotated with Prokka and InterPro.

**Results:**

Although high-risk and sporadic clones carried a similar total number of antimicrobial-resistance genes, 15/67 (22.38%) genes/variants were enriched in high-risk clones, producing class-level enrichment (*p* < 0.002) for aminoglycosides, phenicols, trimethoprim, sulfonamides, and tetracyclines, but not *β*-lactams or fosfomycin. Many resistance determinants are recognized mobile-element cargo such as integron cassettes or plasmid/ICE-borne genes (e.g., *aadA*, *dfrB*, *bla*_VIM-2_, *crpP*, *cmlA*, *floR*), indicating a mobility-linked resistome in high-risk clones. GWAS identified 113 AGEs linked to SK virulence (42 HVA, 71 LVA). HVA regions were enriched for pyoverdine (*fpvA*, *pvdE*, *pvdD*) and LPS O-antigen (*wbpA/B/D*) *loci*, whereas LVA regions were enriched for ICE/conjugation/integrase motifs. *cdsA* and *clpP* were newly associated with *P. aeruginosa* virulence. Phenotypically, high-risk clones were more often strong biofilm producers and none were non-producers. High-risk clones were not consistently more virulent in SK, suggesting success reflects persistence traits (mobile DNA and biofilm under antibiotic pressure).

**Conclusion:**

Accessory-genome GWAS revealed two risk dimensions: acute-virulence programs (HVA) versus mobility functions (LVA) favoring persistence and spread. Because SK measures acute virulence, readouts did not align with high-risk designations. Genomic reports should combine high-risk assignment with accessory-genome and effector profiling to support earlier containment and mechanism-aware, biofilm-focused care.

## Introduction

The dissemination of carbapenem-resistant bacteria has become a serious threat to public health. The ESKAPE pathogens *Enterococcus faecium*, *Staphylococcus aureus*, *Klebsiella pneumoniae*, *Acinetobacter baumannii*, *Pseudomonas aeruginosa* and *Enterobacter* spp. were initially identified as critical multidrug-resistant bacteria capable of evading the effects of antimicrobials (Miller and Arias, 2024). These bacteria often exhibit a multidrug-resistant phenotype and are associated with healthcare-associated infections (HAIs) (Rice, 2008; Mulani et al., 2019). Recent literature has reported the emergence highly virulent and multidrug-resistant isolates, an alarming situation as it limits therapeutic options and causes severe complications in treating infections by such pathogens (Wyres et al., 2020; Lam et al., 2021).

Bacterial pathogenicity is a complex characteristic dependent on various attributes, including the genetic background of the microorganism, each contributing cumulatively to pathogenic potential (Jackson et al., 2011; Allen et al., 2020). Both core genome genes, such as those involved in central metabolism, and accessory genome genes contribute to high levels of virulence (Allen et al., 2020; Panayidou et al., 2020). The success of epidemic bacterial strains, those frequently reported in different geographical regions, is determined by a combination of factors related to pathogenicity and antibiotic resistance (Martínez and Baquero, 2002; Mulet et al., 2013).

Understanding the characteristics behind the success of high-risk clones is crucial for possibly designing specific treatments and aiding in the development of strategies that can be applied by hospital infection control services to minimize the spread of high-risk clones and multidrug-resistant strains in hospital environments. Thus, the central objective of this study was to evaluate factors associated with the success of high-risk clones of *Pseudomonas aeruginosa* through detailed genome analysis of microorganisms selected from a specific clinical setting and those available in public databases. Additionally, we used experimental infection assays with the nematode *Caenorhabditis elegans* to validate hypotheses generated *in silico*. Given the high diversity of the accessory genome in *P. aeruginosa*, comparative genome analysis approaches are powerful tools for identifying new genes associated with the pathogenicity of these isolates. The choice of the infection model in *C. elegans* was based on current ethical restrictions on using murine models when viable alternatives exist, as well as the experimental simplicity and extensive knowledge about the development of *C. elegans* and its response to the virulence of *P. aeruginosa* (Navas et al., 2007; O’Reilly et al., 2014). We aim to combine genotypic and phenotypic characteristics to identify new genomic regions with a central role in the pathogenicity of *P. aeruginosa*.

## Materials and methods

### *Caenorhabditis elegans* and bacterial strains

The *Caenorhabditis elegans* strain used in this study was CF1903-glp-1 (e2144) III obtained from the Caenorhabditis Genetics Center^1^. All strains were maintained on 9 cm plates with Nematode Growth Media (NGM) with *Escherichia coli* OP50 as a food source, following standard methods (Stiernagle, 2006).

*P. aeruginosa* isolates used in this study are clinical isolates listed in (Supplementary Table S1). They were identified by the Microbiology Department of the Clinical Laboratory between 01/2007 and 12/2021, and available in the Clinical Laboratory’s repository at Hospital Israelita Albert Einstein. Species identification as *P. aeruginosa* was performed using MALDI-TOF MS (Matrix-Assisted Laser Desorption/Ionization) (Bruker Daltonics, Billerica, MA, USA). The antimicrobial profile was evaluated using the disk diffusion method for imipenem and meropenem, and the automated Vitek 2 XL System (bioMérieux, France) for ceftazidime, cefepime, amikacin, gentamicin, and ciprofloxacin. Antimicrobial susceptibility results were interpreted according to the Clinical and Laboratory Standards Institute (CLSI) guidelines in effect at the time and, after the implementation of BrCAST/EUCAST, results were interpreted following this protocol. Selected isolates were obtained from blood cultures, bronchial lavage, and tracheal secretion samples. Bacterial samples, which could be reactivated in culture media (MacConkey Agar) and originated from non-repeated patients, were included in this study.

### Endemic level graph construction and selection of isolates

The endemic level of *P. aeruginosa* isolates between 2007 and 2021 was calculated per 10,000 patient-days using the method described by Arantes et al. (2003). Isolates from blood culture, bronchoalveolar lavage, and tracheal secretion samples from all hospital wards were included. Based on the endemic level graph, isolates from or near epidemic moments were selected for genome sequencing. This selection aimed to identify high-risk clones present during these periods and investigate the genetic background of these clones associated with their virulence and persistence in the hospital environment.

### Pulsed-field gel electrophoresis (PFGE)

Genetic relatedness was established using pulsed-field gel electrophoresis (PFGE) as previously described (Pitout et al., 2007). Band patterns for each isolate were manually analyzed and compared using Bionumerics software version 7.1 (Applied Maths, Kortrijk, Belgium). Clustering was performed using the unweighted pair group method with arithmetic mean (UPGMA) with a custom tolerance of 1% and optimization of 1%. DNA relatedness was calculated based on the Dice similarity coefficient, and isolates were considered genetically related if the Dice coefficient was ≥80%, corresponding to possibly related isolates (Pitout et al., 2007).

### Whole genome sequencing (WGS), genome assembly and annotation

Genomic DNA for whole-genome sequencing was isolated as previously described (Salvà-Serra et al., 2018). DNA concentration and purity were assessed using a NanoDrop™ One Spectrophotometer (ThermoFisher Scientific). DNA fragmentation for library construction was performed using the Ion Shear Plus reagents kit, and libraries were constructed with the Ion Plus Fragment Library Kit (ThermoFisher Scientific). Barcoded libraries were quantified using the Bioanalyzer 2,100 and High Sensitivity DNA kit (Agilent, Santa Clara, CA, USA). Libraries were clonally amplified using the Ion PI™ Hi-Q™ Chef Kit (ThermoFisher Scientific) and sequenced with the Ion PI™ Chip Kit v3, Ion 540TM Chip, and Ion PI™ Hi-Q™ Sequencing 200 Kit and Ion 540TM Kit-Chef on the Ion Proton Sequencer and Ion GeneStudio S5. Genome assembly was performed using SPAdes genome assembler software (v3.15.5) with the “iontorrent” and “careful” options (Prjibelski et al., 2020). For genome annotation the Rapid Prokaryotic Genome Annotation (Prokka) was used 1.14.6^2^.

### Genes associated with *Pseudomonas aeruginosa* virulence in *C. elegans*

ABRIcate v.1.0.1^3^ was used to identify virulence genes by screening the Virulence Factor Database (VFDB) (Liu et al., 2018). A gene was considered present if it showed at least 80% identity and coverage (Weiser et al., 2019; Kiyaga et al., 2022). In addition to the VFDB, we used a set of 60 genes known to contribute to the virulence of *P. aeruginosa* against the nematode *C. elegans*. This list of genes was obtained from the study by Vasquez-Rifo et al. (2019). The nucleotide sequences of the 60 genes were retrieved from the Pseudomonas Genome DB^4^. An *in silico* database was created and subsequently added to the ABRIcate pipeline, which conducted a BLASTN search between the database and the target genomes.

### Resistome, serotyping and multilocus sequence typing

The resistome of *P. aeruginosa* isolates was determined using the NCBI Antimicrobial Resistance Gene Finder Plus (AMRFinderPlus) v.3.12.8^5^ with the ‘Core’ and ‘Plus’ databases (Feldgarden et al., 2021). To compare the number of resistance genes between high-risk and sporadic clones, and to test whether the distribution of resistance-gene classes differed between these groups, we used Fisher’s exact test. A *p* < 0.05 was considered statistically significant. All analyses were performed in GraphPad Prism. The capsular serotype (sorotype) of *P. aeruginosa* isolates was determined from the assembly using the *Pseudomonas aeruginosa* serotyper (PAst)^6^. All *P. aeruginosa* assemblies were subjected to Multilocus Sequence Typing (MLST) in silico using the pipeline available at https://github.com/tseemann/mlst, which implements the MLST scheme *for P. aeruginosa* available on PubMLST^7^. For genomes where it was not possible to determine one or more MLST targets using the assembly, the missing genes were amplified by PCR with Platinum™ Taq DNA Polymerase (ThermoFisher Scientific) using the amplification steps and sequencing primers described by Van Mansfeld (van Mansfeld et al., 2009). PCR products were purified and sequenced by Sanger sequencing.

### Multilocus sequence typing (MLST) and selection of high risk and sporadic genomes from the public database

To select *P. aeruginosa* genomes for *in silico* analyses, all genomes of this pathogen available in: NCBI Reference Sequence Database (RefSeq) as of November 2022 were downloaded. At that time, 482 complete genomes, 99 chromosome-level genomes, 4,715 contigs, and 1,953 scaffolds were downloaded, totaling 7,249 *P. aeruginosa* genomes. Metadata associated with these genomes (e.g., isolation date, host, and geographic location) were collected from the National Center for Biotechnology Information (NCBI)^8^ when available. To exclude low-quality or contaminated genomes, genomes downloaded from RefSeq were subjected to a quality assessment step. The CheckM pipeline^9^ was used for this purpose. *P. aeruginosa* genomes with >95% completeness and <5% contamination were retained for further analyses (Diorio-Toth et al., 2022). For typing the 7,249 *P. aeruginosa* genomes and typing of genomes sequenced in this study, the script available at https://github.com/tseemann/mlst was used, employing the MLST scheme for *P. aeruginosa* from the Public Databases for Molecular Typing and Microbial Genome Diversity (PubMLST). To identify high-risk genomes, the definition proposed by Barrio-Tofiño and colleagues and Treepong and colleagues was applied (Treepong et al., 2018; Del Barrio-Tofiño et al., 2020). These studies describe 11 high-risk sequence types (STs): ST111, ST175, ST233, ST235, ST244, ST277, ST298, ST308, ST357, ST395, and ST654.

To minimize sampling bias for specific STs or geographic regions in the public database, genomes classified as sporadic STs (STs not considered high-risk *P. aeruginosa*) were re-sampled to create a “non-redundant” dataset. This re-sampling process was carried out as follows: when two or more genomes shared the same ST, isolation year, host, country of isolation, and, when available, isolation site, only one genome was selected (randomly).

### Pangenome analysis for the determination of the accessory genome of genomes obtained from public databases

The genomes obtained from public databases were annotated using Prokka 1.14.6^10^. The annotation files in gff3 format generated by Prokka were used as input for Roary 3.13.0^11^. The pangenome analysis was performed with the following parameters: -cd 95 (defines the percentage of isolates that must contain a gene for it to be classified as a core genome gene), −i 90 (percentage sequence identity for BLASTp), −s (prevents the addition of paralogous genes). The presence-absence matrix generated by Roary was used as input for Scoary v1.6.16^12^, which was executed with the -collapse parameter, while other parameters were set to default.

### Phylogenetic analysis

Core-genome single nucleotide polymorphisms (cgSNPs) were detected using Snippy v4.6.0^13^, with *P. aeruginosa* PAO1 (GenBank accession number AE004091.2) as the reference genome. A maximum likelihood phylogenetic tree was generated using IQ-TREE v2.2.2.6^14^. ModelFinder identified the best nucleotide substitution model (GTR + F + ASC + R5). Bootstrapping was performed 1,000 times using ultrafast bootstrap (UFBoot). The tree was visualized using iTOL v7 (Letunic and Bork, 2024).

### Core genome, accessory genome and genome comparison for the identification of virulence-associated genomic regions

A core genome of *P. aeruginosa* was defined using SPINE (v0.3.2) (Ozer et al., 2014). The complete genome for each isolate was used as the input to identify sequences present in at least 90% of isolates. AGEnt (v0.3.1) was then used to determine the accessory genome of the isolates sequenced in this study by subtracting the core genome from whole genome (Ozer et al., 2014). ClustAGE was used to determine patterns of shared accessory sequences among different isolates (Ozer, 2018). ClustAGE searches through the multitude of genomic fragments from the accessory genome of every isolate and aligns them to the longest shared contiguous accessory sequence available. We then followed the procedure described by Allen et al. (2020), where shared accessory sequences were referred to as a “bin,” with the longest representative sequence referred to as the “representative bin.” Representative bins were then further processed into subelements based upon alignment breakpoints and contiguous accessory sequences were analyzed for their presence or absence among the isolates.

### Slow killing assays

To evaluate the virulence potential of the *P. aeruginosa* isolates in the *C. elegans* model, the slow killing assay was performed as previously described (Powell and Ausubel, 2008). The *P. aeruginosa* and *Escherichia coli* OP50 strains cryopreserved at −80 °C were routinely revived by streaking onto agar plates to obtain isolated colonies/colony-forming units (CFUs). These plates were incubated overnight at 37 °C. On the following day, a single CFU was transferred to 10 mL of liquid Luria Bertani (LB) medium and incubated overnight in a shaker at 37 °C and 200 rpm. Then, 10 μL of overnight bacterial cultures of the clinical isolates and *Escherichia coli* OP50 (used as a negative control) were spread on 3.5-cm plates containing nematode growth medium (NGM) and incubated at 37 °C for 24 h. Subsequently, the plates were incubated at room temperature for 24 h. Each plate was then seeded with 10 *C. elegans* previously synchronized in the L4 stage, and the plates were incubated at 25 °C to prevent proliferation of the nematodes. The plates were examined daily for 12 days under a dissecting microscope, and the number of surviving worms was recorded. A worm was considered dead when it no longer responded to touch. Each experiment was replicated four times. Bacterial strains were ranked from most to least virulent based on the LT50 (time at which 50% lethality was observed). Differences in *C. elegans* survival rates were determined using the log-rank test (GraphPad Prism).

### Association of genetic elements with virulence

To investigate the association of subelements with virulence we followed the protocol proposed by Vasquez-Rifo et al. (2019). The Mann–Whitney (MW) ranking test and linear regression (LR) analysis were applied to test the association of the presence/absence of every subelement with virulence, defined by LT50 (time at which 50% lethality was observed). If both tests yielded a *p-*value lower than 0.05, and at least one of the tests yielded a *p-*value smaller than 0.01 the subelement was considered associated with virulence. Subelements with negative slope (based on linear regression) were associated with high virulence (high-virulence associated or HVA), while subelements with positive slope were associated with low virulence (low-virulence associated or LVA).

All the *p* values are shown in log10 scale as absolute values. The control for multiple hypothesis testing was performed using a permutation test as previously described^15^ (Vasquez-Rifo et al., 2019). Briefly, ten thousand permutations of the virulence values (LT50) and their assignment to strains were generated, and the MW and LR association tests were repeated for each permutation. For each subelement, the number of times that it received a better *p*-value using the shuffled virulence data compared to the original one was recorded, separately for MW and LR. The above count was divided by 10,000 to obtain the permutation corrected *p-*value for the MW and LR tests. The MW and LR *p-*values were considered significant if at least for one of the corresponding corrected *p-*value was lower than 0.05.

For subelements that passed the multiple-hypothesis testing, coding sequences (CDSs) were annotated with Prokka. The resulting protein sequences predicted by Prokka were then submitted to InterPro^16^ to assist and refine CDS functional annotation (Blum et al., 2025).

### Biofilm formation assay

Biofilm quantification was performed in 96-well plates as described by O'toole (2011). Briefly, a culture of a *P. aeruginosa* isolate grown overnight in LB at 200 rpm was diluted 1:100 in fresh LB broth. Then, 100 μL of the dilution was added to the wells of a 96-well microplate, in quintuplicate for each strain evaluated, and the microplate was incubated at 37 °C for 24 h. Planktonic cell growth was removed by washing three times with distilled water. The biofilm was stained with 125 μL of 0.1% crystal violet (CV) for 15 min, washed four times with distilled water, and the plate was left to dry for a few hours until complete evaporation of the water. Subsequently, the retained CV was solubilized with 125 μL of 30% acetic acid and measured using a Varioskan LUX plate reader (Thermo Fisher Scientific) at 550 nm absorbance. Wells in which the strains had an OD greater than the blank were considered to have biofilm production. Acetic acid was used as the blank. Next, we calculated the cutoff values using the following formula: OD control (ODc) = average OD of the negative control + (3 × standard deviation (SD) of the negative control), OD of the strain = average OD of the strain − ODc. The results were interpreted within four categories: OD ≤ ODc: no biofilm production (NBP); ODc < OD ≤ 2 × ODc: weak biofilm production (WBP); 2 × ODc < OD ≤ 4 × ODc: moderate biofilm production (MBP) and 4 × ODc < OD: strong biofilm production (SBP).

### Data availability

All genome sequences were deposited in the GenBank BioProject: PRJNA1275064 and accession numbers are also available in Supplementary Table S2.

## Results

### Prevalence and selection of *P. aeruginosa* isolates for genome comparisons

Between January 2007 and December 2021, 4,806 unique *P. aeruginosa* isolates were identified, as reported by the clinical laboratory. These isolates were obtained from three sources: blood cultures, bronchial lavage, and tracheal secretions. The most predominant site was tracheal secretions, accounting for 76.05% of the samples, followed by blood cultures at 14.71% and bronchial lavage at 9.23%. Carbapenem resistance (imipenem and/or meropenem) was observed in nearly 60% of the isolates.

**Figure 1:**
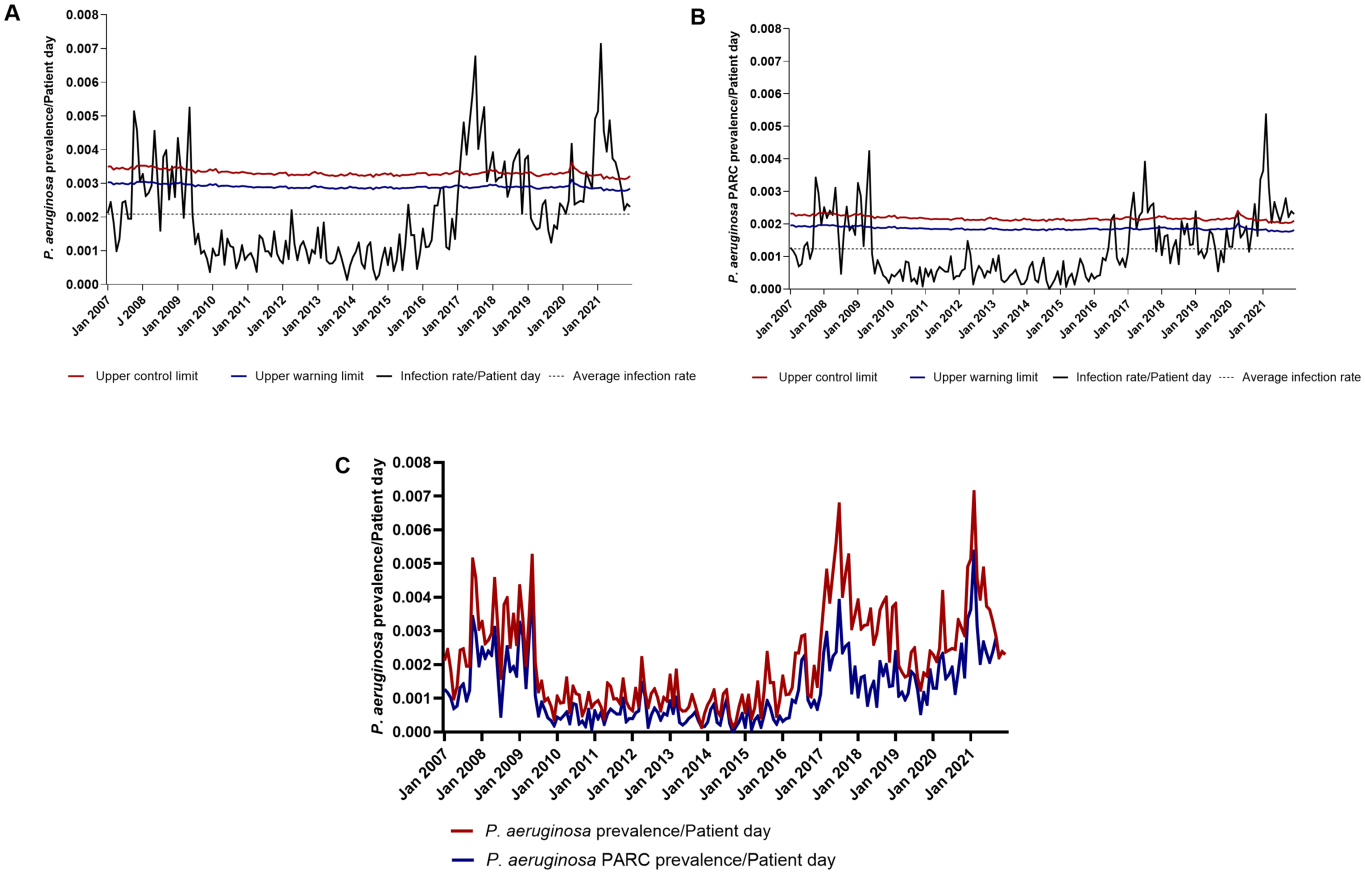
Prevalence of *P. aeruginosa* over time. **(A)** Prevalence of *P. aeruginosa* resistant or susceptible to carbapenems. **(B)** Prevalence of *P. aeruginosa* resistant to carbapenems. **(C)** Comparison of the prevalence rate of *P. aeruginosa* susceptible or resistant to carbapenems vs. the prevalence rate of *P. aeruginosa* isolates resistant to carbapenems (*P. aeruginosa* CR).

Based on the distribution data, two prevalence graphs for *P. aeruginosa* were constructed: one considering all 4,806 isolates reported between 2007 and 2021 (Figure 1A) and another focusing only on the subset of carbapenem-resistant *P. aeruginosa* isolates (Figure 1B). A comparison of the prevalence trends for all 4,806 isolates and the 2,834 carbapenem-resistant isolates demonstrated that the resistant isolates followed the general prevalence trend of the overall *P. aeruginosa* population (Figure 1C).

**Figure 2:**
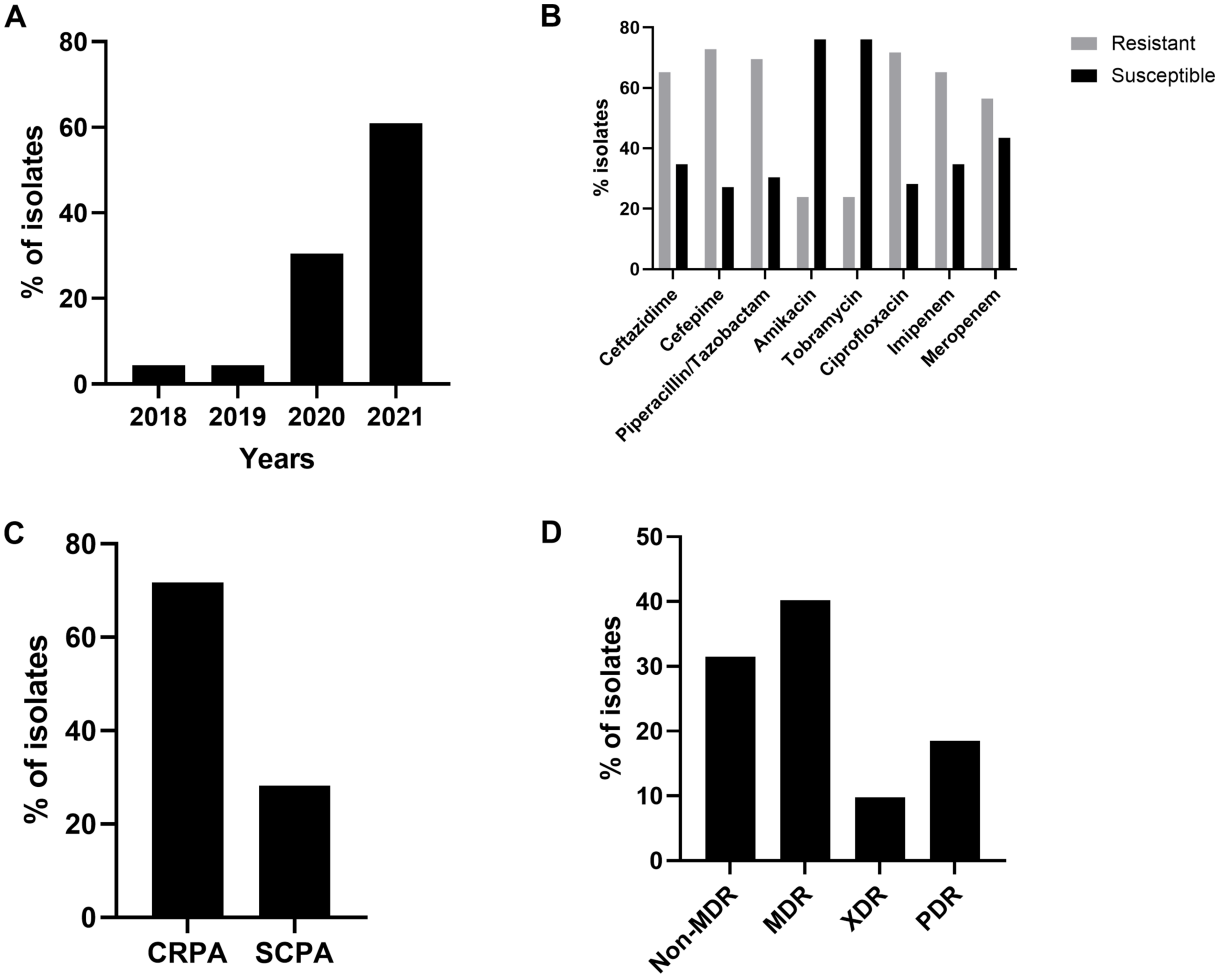
Characteristics of the 92 *P. aeruginosa* blood culture Isolates selected for genome comparisons. **(A)** Distribution of isolates between 2018 and 2021. **(B)** Susceptibility profile to the tested antimicrobials. **(C)** Percentage susceptibility profile to the carbapenem class. **(D)** Percentage of isolates classified as Non-MDR, MDR, XDR, and PDR.

For genome comparisons, isolates were selected near or during epidemic periods (Figures 1A,B) aiming to identify high-risk clones present during these periods and investigate genetic markers associated with virulence and persistence in the hospital. During the reactivation process, it was observed that older isolates were often nonviable. Consequently, more recent isolates, collected between 2018 and 2021, were selected for molecular analyses. Additionally, isolates from blood cultures were prioritized due to their higher likelihood of association with healthcare-associated infections. This final selection resulted in a set of 124 *P. aeruginosa* isolates, of which 74.19% (92/124) were successfully reactivated. These 92 isolates exhibited the following characteristics: 60.86% (56/92) were reported in 2021 (Figure 2A), the isolates demonstrated resistance to most tested antibiotics, except amikacin and tobramycin (Figure 2B), nearly 72% of the isolates were resistant to carbapenems (imipenem and/or meropenem) (Figure 2C), and approximately 10% of the isolates were classified as pan-resistant (PDR) (Figure 2D).

For this set of isolates, libraries were prepared for sequencing, followed by subsequent analyses of the virulome, resistome, and additional assays using the *C. elegans* model.

### Characterization of the *P. aeruginosa* population by PFGE

**Figure 3:**
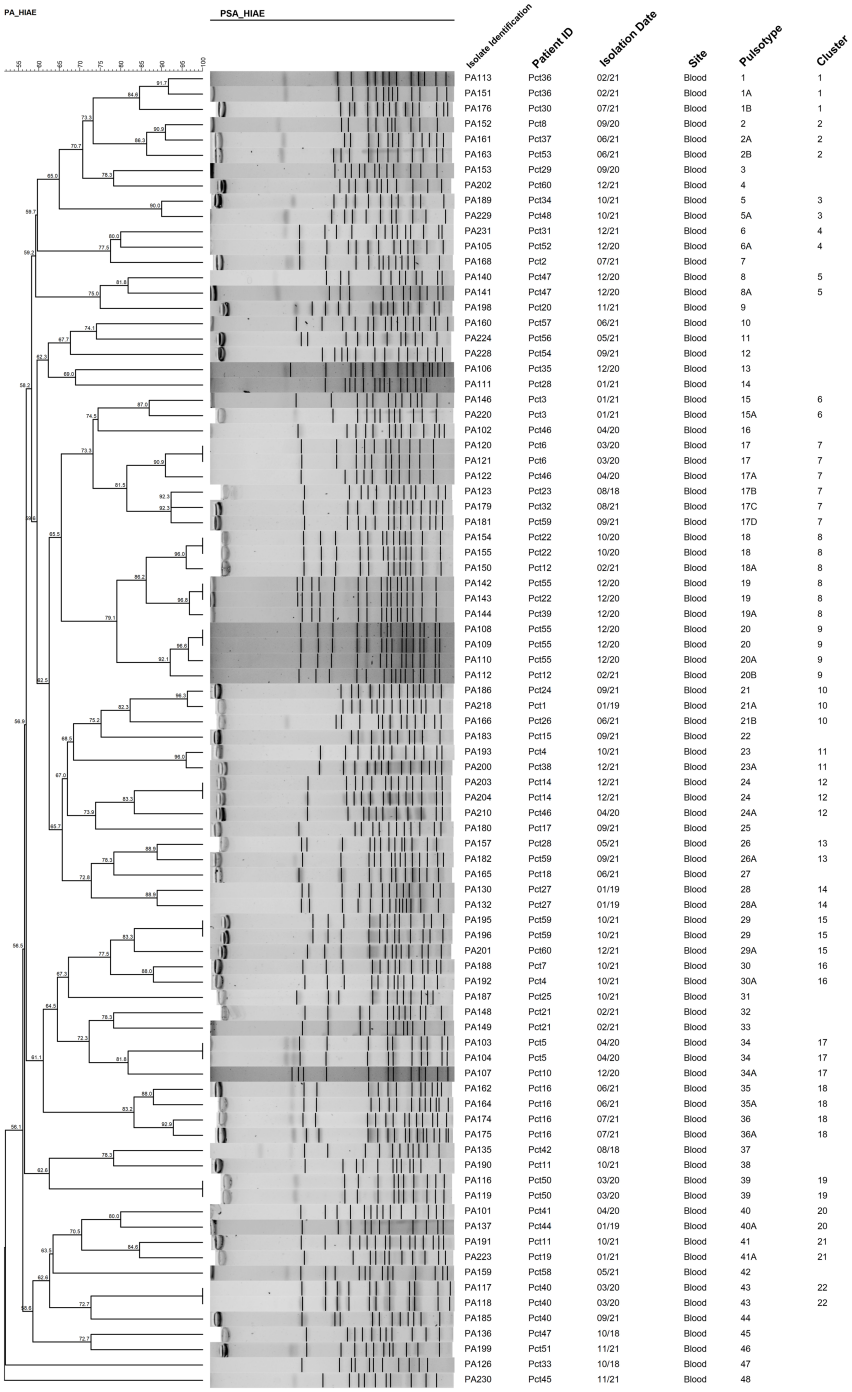
PFGE-based relationships among the 86 *P. aeruginosa* isolates selected from periods of higher incidence. Isolate Identification: Identification of each isolate. Patient ID: Unique number assigned to each patient. Isolation Date: (Month/Year). Site: Isolation site. Pulsetype: PFGE profile of the isolate based on the banding pattern. Cluster: Two or more isolates sharing ≥80% similarity based on the banding pattern. The dendrogram was generated by BioNumerics v7.5 software.

Of the 92 *P. aeruginosa* blood culture isolates, population characterization using PFGE profiles was successfully performed for 93.47% (86/92) of the isolates. The 86 typed isolates were grouped into 48 different pulsetypes based on their banding patterns (Figure 3). Additionally, 72.09% (62/86) of the isolates were clustered into 22 groups (≥2 isolates with ≥80% similarity). These results indicate that the 86 evaluated *P. aeruginosa* isolates represent a highly heterogeneous population.

### Molecular typing and phylogenetic analysis of *P. aeruginosa* isolates

For sequencing, one isolate per pulsetype was selected. Of the 86 isolates subjected to PFGE, 66 (76.74%) were successfully sequenced and assembled. To enhance our genomic analysis, an additional set of 18 *P. aeruginosa* strains was included: 13 strains previously sequenced by our research group (Brüggemann et al., 2018), and 5 additional strains from blood cultures, collected in 2015, 2016 and 3 reported in 2021. All newly assembled genomes are available at NCBI BioProject: PRJNA1275064 and accession numbers are available in Supplementary Table S2.

**Figure 4:**
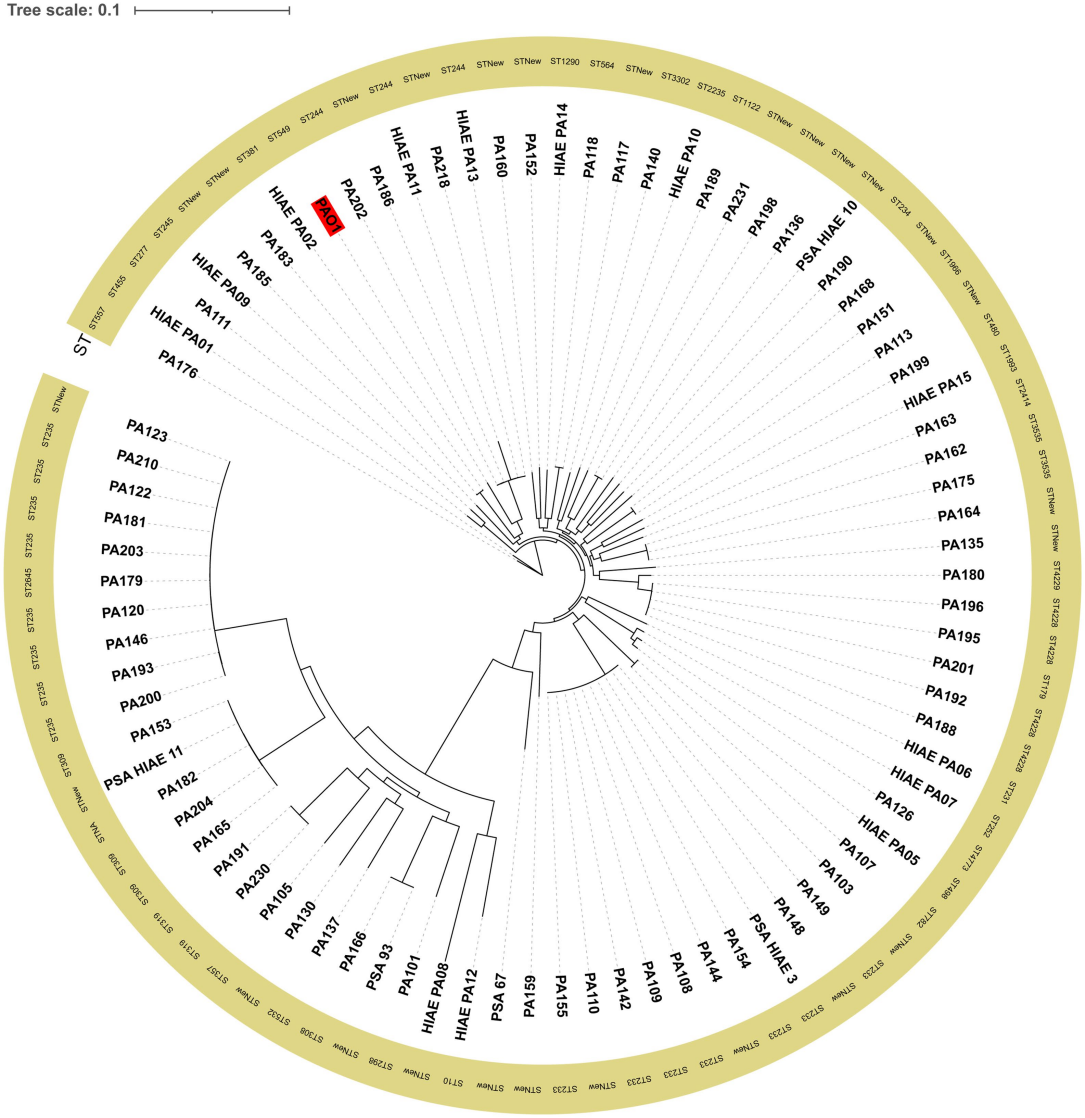
Maximum likelihood tree generated from cgSNP alignment of *P. aeruginosa* isolates. PAO1, highlighted in red, was used as reference in the tree. ST: Sequence type.

The MLST analysis grouped the 84 *P. aeruginosa* strains into 35 distinct STs, clustering 57 strains, while another 26 strains carried a new combination of MLST target *loci* not yet present in the pubMLST database and these were classified as STNew for this study. One strain (PA182) could not be typed by MLST due to the loss of one of the target genes and it was not included when comparisons were made between high-risk and sporadic clones. The predominant sequence types among the known STs were ST235 (8/83; 9.63%) and ST244 (7/83; 8.43%). The clustering generated by the cgSNP alignment corresponded to the MLST ST assignment of the strains (Figure 4). The cgSNP analysis showed that the 84 *P. aeruginosa* strains had between 6,114 and 17,152 cgSNPs when compared to the reference strain PAO1. The strain with the highest number of cgSNPs was HIAE_PA08, with 17,152.

### Resistome of *P. aeruginosa*

**Figure 5:**
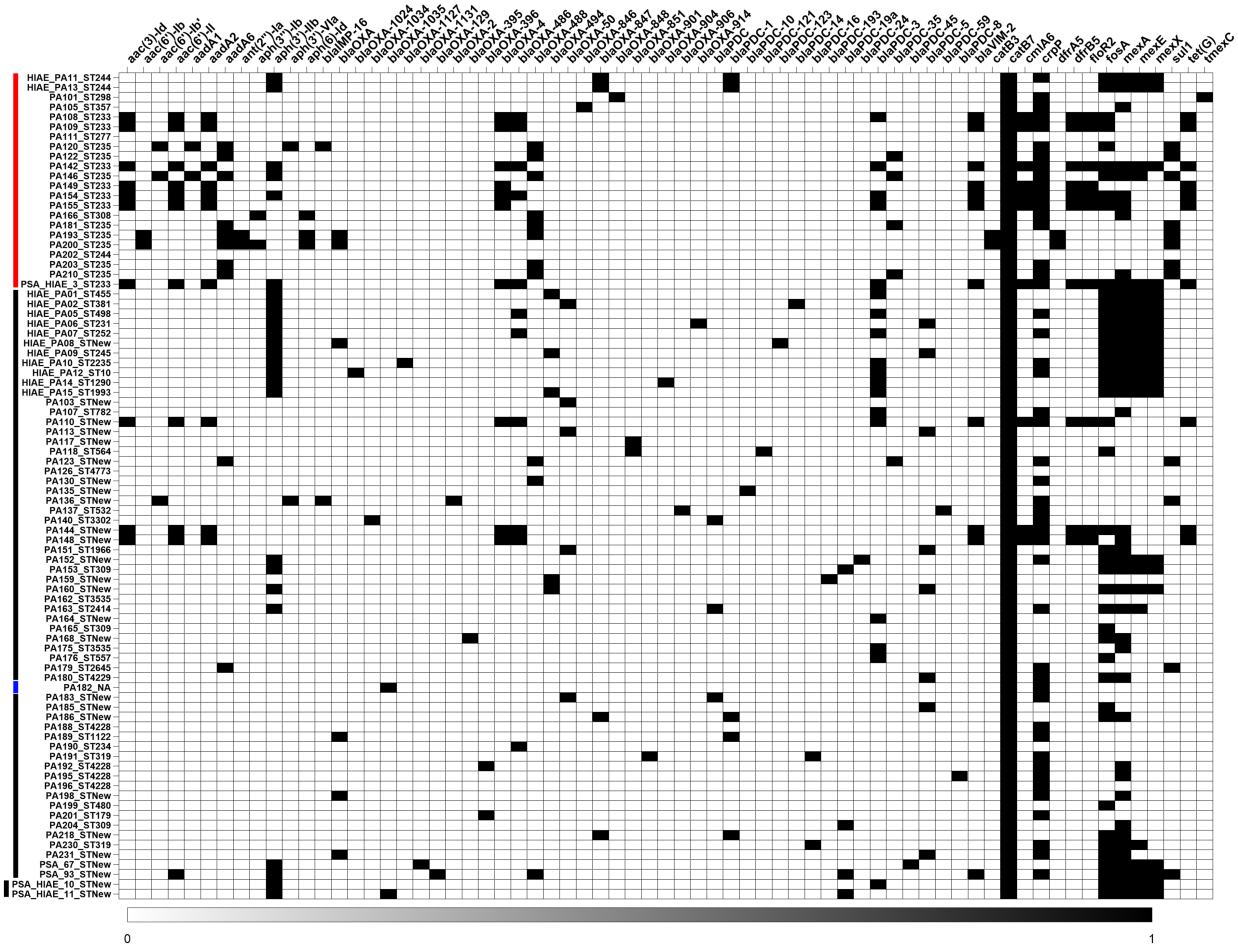
Genes and gene variants identified in *P. aeruginosa* sequenced in this study. White cells: absence, Black cells: presence. The vertical red line before the strain IDs highlights the strains belonging to high-risk clones, while the black line highlights the clones that do not belong to the high-risk group, the blue line highlights the strain without ST (NA: Not assigned).

We analyzed the resistome of the 84 sequenced *P. aeruginosa* strains. This analysis revealed that these strains carried 67 unique genes or variants related to antimicrobial resistance, which confer resistance to eight antibiotic classes: aminoglycosides, beta-lactams, phenicols, fluoroquinolones, trimethoprim, fosfomycin, sulfonamides, and tetracyclines (Figure 5 and Supplementary Table S3).

In *P. aeruginosa*, horizontal gene transfer predominantly affects two antibiotic classes (aminoglycosides and *β*-lactams) but has also been described for other antimicrobial agents (Breidenstein et al., 2011). Aminoglycosides constituted the second most frequent class of resistance genes or variants (17.91%). Twelve genes were identified, with the primary representatives being *aph(3′)-IIb* (29.76%; 25/84), *aac(6′)-Il* (13.10%; 11/84), and *aac(3)-Id*, *aadA2*, and *aadA6*, each at 11.90% (10/84).

The beta-lactam class had the highest number of resistance genes or variants (61.19%), with 41 genes or variants identified. Notably, the genes *bla*_VIM-2_ (13.10%; 11/84) and *bla*_IMP-16_ (2.38%; 2/84) were present, both of which confer resistance to beta-lactams and are among the key genes disseminated in Latin America (Escandón-Vargas et al., 2017).

An investigation into the resistance phenotype of the 84 sequenced strains showed that 71.43% of the population exhibited a multidrug-resistant (MDR, XDR, or PDR) phenotype, as previously defined by Magiorakos et al. (2012). This finding aligns with the diversity of antimicrobial resistance (AMR) genes or variants identified in these strains. The number of AMR genes per isolate ranged from 1 to 18.

Overall, when we compared high-risk and sporadic clones, both groups carried a similar total number of AMR genes. However, when we tested each gene/variant individually between high-risk versus sporadic groups, we found that 15 of the 67 identified genes/variants (22.38%) were significantly more frequent among high-risk clones (Fisher’s exact test, *p* < 0.05). These 15 were: aminoglycosides *aac(3)-Id*, *aac(6′)-Il*, *aadA2*, *aadA6*, *aph(6)-Id*; β-lactamase/carbapenem-related *bla*_OXA-4_, *bla*_OXA-488_, *bla*_PDC-35_, *bla*_VIM-2_; phenicols *cmlA6*, *floR2*; fluoroquinolones *crpP*; trimethoprim *dfrB5*; sulfonamides *sul1*; and tetracyclines *tet(G)*. At the drug-class level, significant enrichment in high-risk clones was observed for aminoglycosides (*p* < 0.0001), phenicols (*p* = 0.0003), fluoroquinolones (*p* = 0.0236), trimethoprim (*p* = 0.0002), sulfonamides (*p* = 0.0019), and tetracyclines (*p* = 0.0012); by contrast, β-lactams (*p* = 0.0930) and fosfomycin (*p* = 0.6197) showed no statistically significant difference (Fisher’s exact test). Although we did not resolve genomic context, several AMR determinants enriched in high-risk clones are typically associated with mobile genetic elements: *aadA2*, *aadA6* and *dfrB5* (class-1 integron cassettes), *sul1* (3′-conserved segment), *bla*_VIM-2_ (frequently integron-associated), *crpP* (often plasmid/ICE-borne), and *cmlA6* and *loR2* (commonly plasmid/integron-linked). This suggests a mobility-linked resistome profile in high-risk lineages.

### Virulome of *P. aeruginosa*

**Figure 6:**
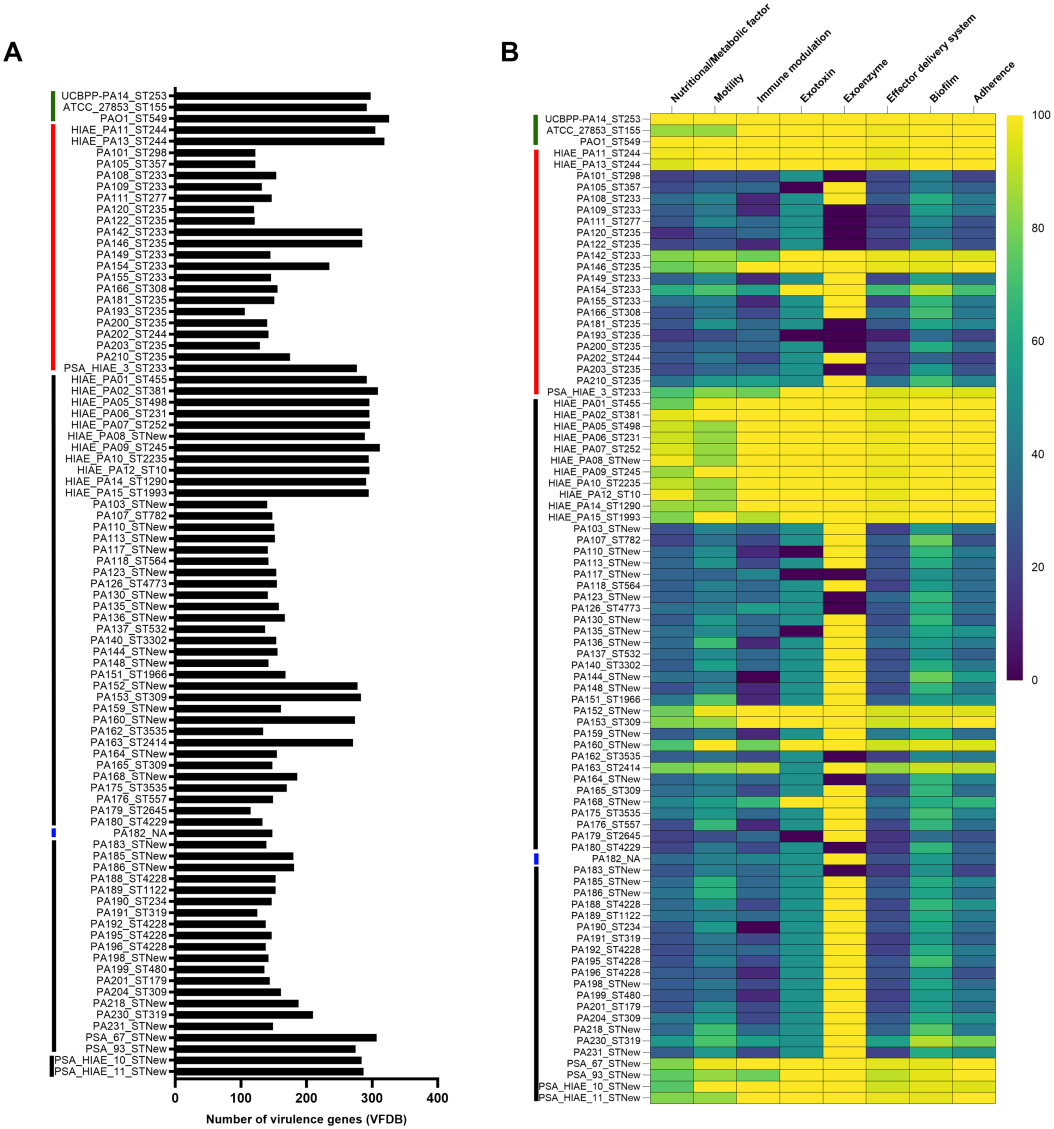
Number of virulence genes identified through VFDB and their classification into virulence classes. **(A)** Number of virulence genes per strain. **(B)** Virulence classes and number of genes belonging to each class. The vertical red line before the strain IDs highlights the strains belonging to high-risk clones, the black line highlights the clones that do not belong to the high-risk group, the blue line identifies strain without ST (NA: Not assigned), and the green line identifies the reference strains (UCBPP-PA14, PAO1 and ATCC_2785).

The adaptability and flexibility of *P. aeruginosa* are attributed to its extensive array of virulence factors, which enable it to tailor its response to various environmental stressors (Jurado-Martín et al., 2021). The 84 *P. aeruginosa* strains analyzed in this study underscore the pathogen’s high adaptability and flexibility, as the number of virulence factors identified in these strains ranged from 106 to 319 (Figure 6A). Eight categories of virulence factors were identified: effector delivery system, nutritional/metabolic factors, motility, adherence, biofilm, immune modulation, exotoxins, and exoenzymes. The most represented categories were effector delivery system (34.86%), nutritional/metabolic factors (16.51%), motility (16.21%), and adherence (14.07%).

When dividing the 83 *P. aeruginosa* strains with ST sequenced in this study into high-risk clones (*n* = 22) and sporadic clones (*n* = 61) and comparing which clone group carried a higher percentage of virulence genes, we identified that strains belonging to sporadic clones frequently carried the highest percentage of virulence genes, 61.16% (200/327), while high-risk clones carried 21.71% (71/327), and 17.13% (56/327) of the genes had the same distribution in both groups.

Due to the importance of UCBPP-PA14 as a highly virulent strain in different test models, including the nematode *C. elegans* (Lewenza et al., 2014; Scott et al., 2019; Wang et al., 2021; Grace et al., 2022), a comparison was made between the virulome of the UCBPP-PA14 strain (NCBI RefSeq assembly: GCF_000014625.1) and all 84 strains sequenced in this study. Additionally, the reference strains PAO1 (NCBI RefSeq assembly: GCF_000006765.1) and ATCC_2785 (NCBI RefSeq assembly: GCF_001086625.1) were included.

The UCBPP-PA14 strain harbored 298 virulence genes according to the VFDB database (Figure 6A). Among these genes, 56 (18.79%) were found in all the strains analyzed in this study (the 84 sequenced strains and the three reference strains: UCBPP-PA14, PAO1, and ATCC_2785). The 242 virulence genes present in UCBPP-PA14 but with irregular distribution among the other strains were further investigated and grouped by class to assess how similar the virulome of the other strains was to UCBPP-PA14. These 242 genes were categorized into 8 virulence classes (Figure 6B). Among these classes, the exoenzyme class was present in more than 80% of the strains (81.61%), making it the most conserved class when compared to UCBPP-PA14 (Figure 6B). However, when we analyzed only the high-risk clones for the presence of the exoenzyme class, we found that 59.09% (13/22) of the high-risk clones were positive, compared to 88.52% (54/61) of the sporadic clones. This class of virulence factors plays an important role in the immune evasion of *P. aeruginosa*, as it locally suppresses the host immune response, creating a favorable environment for colonization and the establishment of chronic infection (Kharazmi, 1991).

**Figure 7:**
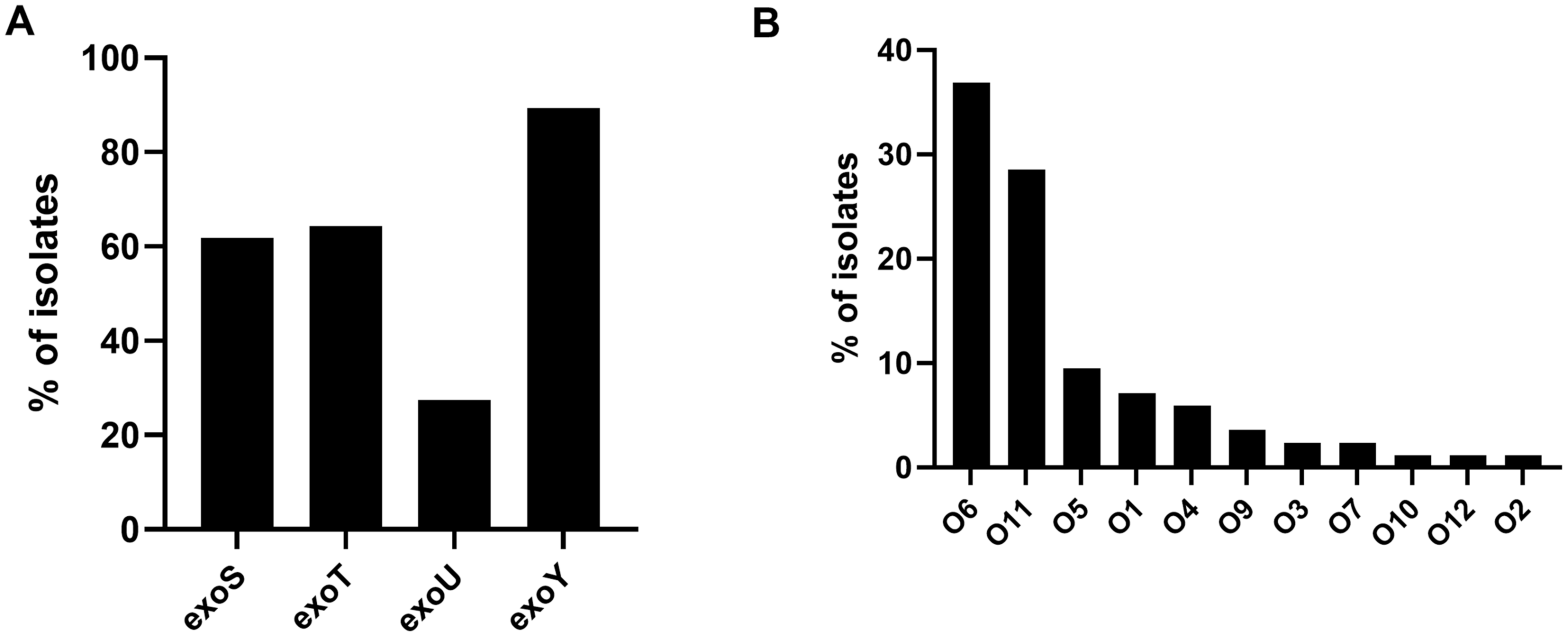
Distribution of Type III Secretion System genes and serotypes in *P. aeruginosa* sequenced genomes. **(A)** Distribution of each gene of the Type III Secretion System. **(B)** Distribution of the serotypes identified in the strains of this study.

During acute disease, *P. aeruginosa* utilizes the toxins of the type III secretion system (T3SS) to evade the host immune system and establish infection. For this reason, we analyzed the presence of T3SS components in the strains studied and identified the following distributions: *exoY*: 89.29%, *exoT*: 64.29%, *exoS*: 61.90%, and *exoU*: 27.38% (Figure 7A). Of the four exotoxins (ExoS, ExoT, ExoU, ExoY), ExoU, a potent phospholipase that disrupts the plasma membrane, leading to rapid cell death, is associated with high virulence (Treepong et al., 2018). The distribution of serotypes among the 84 *P. aeruginosa* strains revealed 11 different serotypes: O6, O11, O5, O1, O4, O9, O12, O3, O7, O10, and O2. The most prevalent serotypes were O6 (*n* = 31; 36.90%), O11 (*n* = 24; 28.57%), and O5 (*n* = 8; 9.52%) (Figure 7B). Notably, serotypes O6 and O11 account for approximately 50% of *P. aeruginosa* strains circulating worldwide (Nasrin et al., 2022). Although this study identified that the strains belonged to 11 different serotypes, their distribution was not uniform between high-risk clones and sporadic clones. High-risk clones comprised only 4 serotypes O11, O6, O5, and O12, with O11 and O6 being the most prevalent in this group, at 50 and 31.81%, respectively. In contrast, 10 serotypes were found among sporadic clones, with O6 and O11 also being the most common in this group, at 39.34 and 19.67%, respectively.

### Virulence toward *C. elegans* strongly varies among *P. aeruginosa* strains

**Figure 8:**
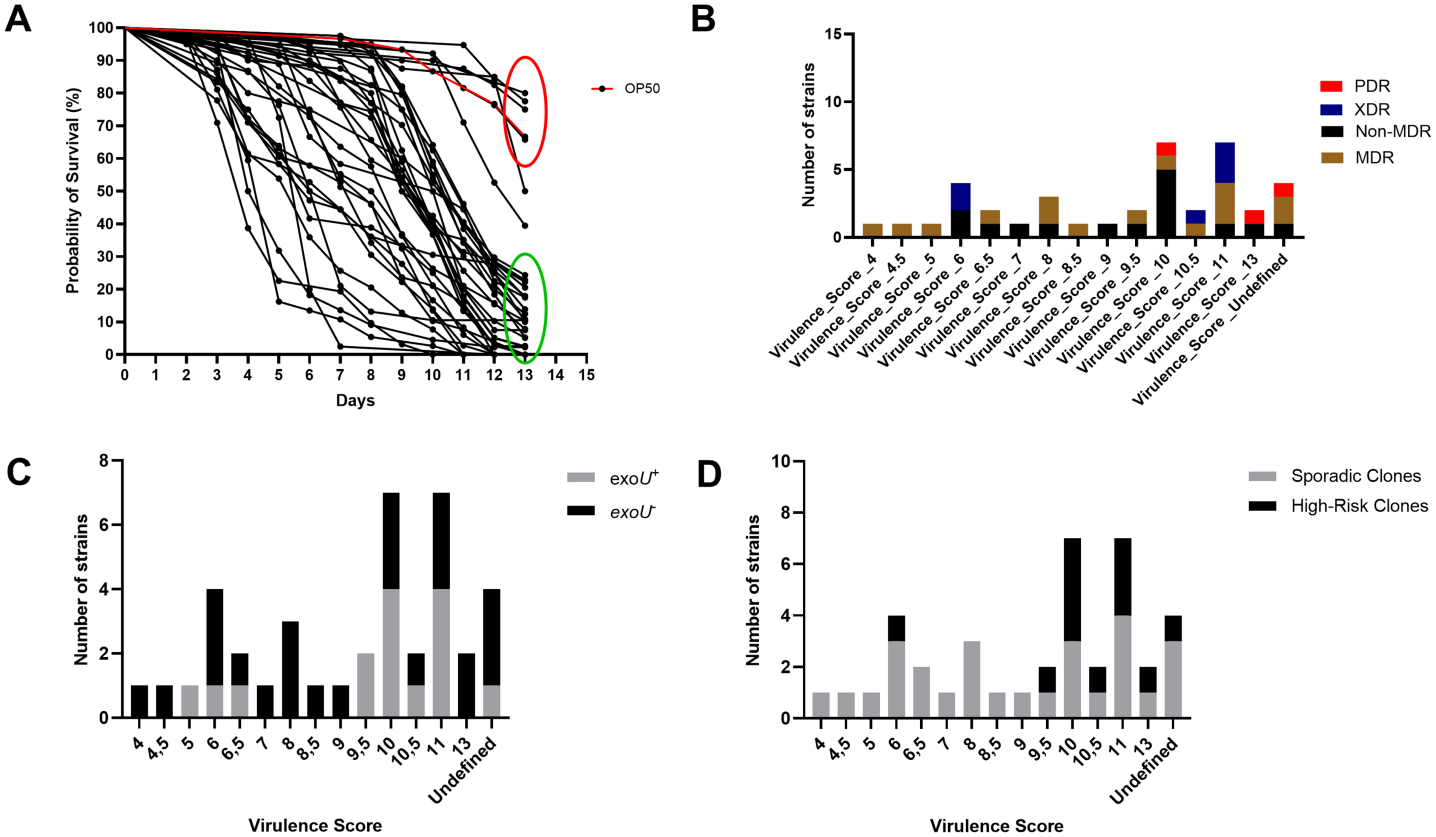
Distribution *P. aeruginosa* virulence toward *C. elegans*. **(A)** Survival curves of *C. elegans* exposed to the studied collection of 39 *P. aeruginosa* strains. The red circle highlights the least virulent strains, with a profile like OP50, and the green circle the most virulent ones. **(B)** Virulence Score and antimicrobial susceptibility phenotype: Non-multidrug-resistant (Non-MDR), multidrug-resistant (MDR), extensively drug-resistant (XDR) and pandrug-resistant (PDR). **(C)** Virulence Score and presence/absence of *exoU*. **(D)** Distribution of Virulence Scores among high-risk and sporadic clones.

Interactions of *P. aeruginosa* with *C. elegans* were assessed for a set of 39 *P. aeruginosa* strains. The analysis obtained from the survival curve demonstrates the formation of two main groups: one more virulent and the second group consisting of strains with a profile like OP50 (Figure 8A). The virulence score ranged from 4 (highly virulent) to undetermined (i.e., if the probability of survival exceeds 50% at the longest time point, then the median survival time cannot be computed). Scores 10 and 11 were the most common among the evaluated strains, each representing 17.94% (7/39). Strains considered as most virulent were also multidrug resistant (Figure 8B). ExoU is one of the main virulence markers in *P. aeruginosa* (Treepong et al., 2018). Among the evaluated strains, those carrying the *exoU* gene showed varying virulence scores (Figure 8C). The 39 *P. aeruginosa* strains varied significantly in their virulence scores; we investigated how this distribution differed between high-risk and sporadic clones (Figure 8D). The high-risk clones (30.76%; 12/39) were spread across 7 different virulence scores, with score 6 representing the highest virulence. The most virulent strains were associated with sporadic clones, specifically ST455, and two strains with sequence types not previously reported.

**Figure 9:**
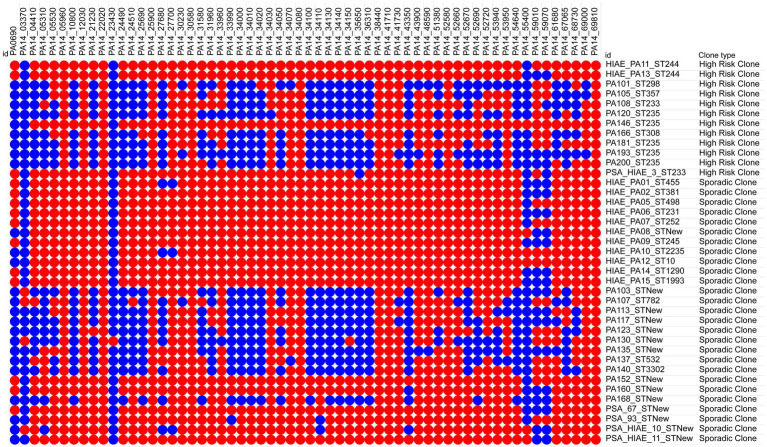
Presence-absence matrix of the 60 virulence genes associated with *P. aeruginosa* infection in *C. elegans*. Blue: absence of the gene. Red: presence of the gene.

After performing the virulome analysis, a collection of 60 virulence genes previously identified in *P. aeruginosa* contributing to the virulence of this pathogen toward *C. elegans* was investigated (Bartell et al., 2017; van Tilburg Bernardes et al., 2017; Vasquez-Rifo et al., 2019). The strains carried between 25 and 95% of the 60 genes analyzed (Figure 9).

Among these 60 genes, 10 genes (PA14_05960/cold-shock protein, PA14_12030/hypothetical protein, PA14_22020/cell division inhibitor MinD, PA14_25900/fabV, PA14_30580/LuxR family transcriptional regulator, PA14_34080/ Lip3, PA14_38440/citronelloyl-CoA dehydrogenase, GnyD, PA14_41710/hypothetical protein, PA14_52580/aspartate kinase, and PA14_69810/nitrogen regulatory protein P-II 2) were identified in all strains. Studies have shown that mutations in these genes lead to attenuation of virulence toward *C. elegans* (Mou et al., 2011; Feinbaum et al., 2012).

On the other hand, the genes with the lowest frequency among the 39 strains evaluated were PA14_23430/hepP (1/39), PA14_03370/hypothetical protein (4/39), and PA14_55400/hypothetical protein (6/39). It has been demonstrated that a mutation in PA14_23430/hepP in strain UCBPP-PA14 resulted in its inability to kill *C. elegans* (Dzvova et al., 2017). Additionally, PA14_03370/hypothetical protein and PA14_55400/hypothetical protein contribute to the virulence of *P. aeruginosa* against *C. elegans* (Lee et al., 2006; Faure et al., 2014). Among the high-risk clones, the genes PA14_03370 (hypothetical protein) and PA14_23430 (hepP) were absent in all isolates, while they were detected in 14.81 and 3.70% of the sporadic clones, respectively. Overall, sporadic clones carried a higher proportion of the 60 virulence genes previously identified in *P. aeruginosa* as contributing to its pathogenicity in *C. elegans* (Bartell et al., 2017; van Tilburg Bernardes et al., 2017; Vasquez-Rifo et al., 2019). However, high-risk clones carried 11 genes that were present in all isolates within this group. Of these, 10 were also found in all sporadic clones. The exception was PA14_27700 (transcriptional regulator), found in 88.89% of sporadic clones.

### *Pseudomonas aeruginosa* virulence correlates with the presence of accessory genome elements

We performed a genome association analysis to test whether the virulence of *P. aeruginosa* strains toward *C. elegans* could be associated with the presence or absence of accessory-genome elements (AGEs), or subelements, other than genes evaluated in the previously section. In this analysis, virulence was defined as a quantitative trait for each strain, corresponding to the score obtained from the *C. elegans* survival assay when fed each strain. The association between AGEs and virulence was measured using the Mann–Whitney (MW) test and linear regression (LR), followed by a permutation approach to control for multiple statistical tests and assess the reliability of the *p*-value.

**Figure 10:**
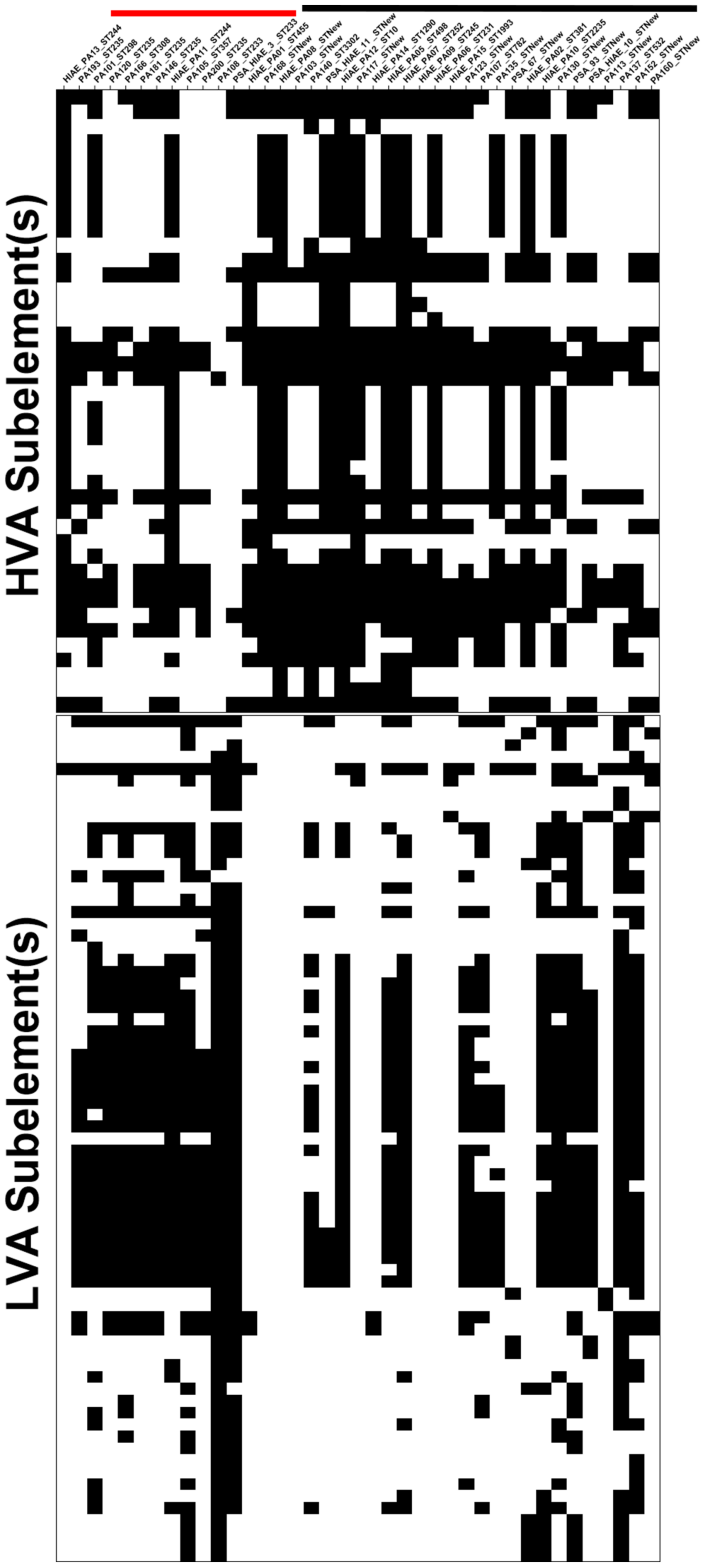
Association between *P. aeruginosa* accessory genome subelements and bacterial virulence. Presence/absence matrix for HVA subelements **(top)** and LVA subelements **(bottom)**. Black cells: presence of subelement. White cells: absence of subelement. The horizontal red line before the strain IDs highlights the strains belonging to high-risk clones, the black line highlights the clones that do not belong to the high-risk group.

In the association analysis, we evaluated 10,899 subelements associated with the accessory genome of the 39 *P. aeruginosa* isolates analyzed. Among these, we identified 113 subelements associated with virulence, either positively or negatively (*p*-value < 0.01 for the MW or LR test, Figure 10) (Supplementary Table S4). Forty two of the 113 subelements (37.16%) were associated with highly virulent strains and were referred to as HVA (high-virulence-associated) subelements, and 71 subelements (62.83%) were associated with low-virulence strains and were referred to as LVA (low-virulence-associated) subelements.

We compared which clone groups (high-risk clones vs. sporadic clones) carried a higher proportion of HVA or LVA elements. Among the 42 subelements that contributed positively to virulence in *C. elegans*, all were found in greater proportion in sporadic clones than high-risk clones; only 2.38% (1/42) were more frequently found in high-risk clones. In contrast, among the subelements that contributed negatively to virulence, 97.18% (69/71) were more commonly present in high-risk clones than in sporadic clones.

Of all 113 subelements used for annotation, Prokka was able to identify one or more coding sequences (CDS/CRISPR) in 57 subelements (Supplementary Table S5). These 57 subelements generated 120 CDSs; two subelements were annotated as CRISPR, 102 were annotated as hypothetical proteins. The nucleotide sequences of the 120 CDSs generated by Prokka were submitted to a BLASTN search using the core_nt nucleotide database, selecting “*Pseudomonas aeruginosa* (taxid:287)” as the organism, and we performed the same analysis using only the genome of the UCBPP-PA14 strain. In the first approach, after the BLASTN analysis, only 22 CDSs remained annotated as hypothetical proteins (Supplementary Table S5). In the second comparison, using only the genome of the UCBPP-PA14 strain, 74 CDSs aligned with genes or gene fragments from the UCBPP-PA14 strain (Supplementary Table S5). As a complementary approach, using InterPro, we were able to annotate 73 of 120 CDSs (Supplementary Table S5).

### HVA-associated genes, LVA-associated genes, and their functional roles

Among the identified HVA subelements, some were previously recognized as key virulence factors in *P. aeruginosa*, particularly those related to pyoverdine biosynthesis, an important virulence factor: PA14_33690 (*pvdE*), PA14_33680 (*fpvA*), PA14_33650 (*pvdD*), PA14_33610 (peptide synthase), and PA14_33630 (pvdJ). Other HVA genes included *wzzB*, *wbpA*, *wbpB*, *wbpD*, *wbpE*, and *wzy*, which encode enzymes required for LPS O-antigen synthesis, a structural component of the bacterial outer membrane (Maldonado et al., 2016). These genes are already known to contribute to *P. aeruginosa* virulence.

Our analysis also revealed some genes not previously linked to virulence toward *C. elegans* (Supplementary Table S5). For example, *cdsA* (PA14_17120/phosphatidate cytidylyltransferase) is involved in glycerophospholipid metabolism, which plays a key role in bacterial adaptation to environmental changes and bacteria-host interactions (Kondakova et al., 2015), and *clpP* that has not been previously associated with *P. aeruginosa virulence* toward *C. elegans* but has been linked to biofilm formation in other pathogenic species and mutations in *clpP* in *P. aeruginosa* resulted in reduced biofilm formation and swarming defects (Fernández et al., 2012; Zheng et al., 2020). Also, *wecA*, involved in LPS biosynthesis, has been linked to virulence, similar to *wbpX* (PA5449), *wbpY* (PA5448), and *wbpZ* (PA5447) (Abeyrathne et al., 2005), and *gnu*, an enzyme involved in O-antigen biosynthesis that contains a conserved domain from the WcaG protein family. WcaG is involved in capsular fucose synthesis and enhances bacterial resistance to phagocytosis by macrophages (Derakhshan et al., 2016).

Several genes associated with low virulence (LVA) were annotated as integrative conjugative elements (ICEs), such as PA14_60130, PA14_59660, PA14_59670, PA14_59680, PA14_59690, and PA14_59860. Some were also linked to phages, including bin5_se00011, bin19_se00022 and bin19_se00012 (Supplementary Table S5). Additionally, CRISPR was identified as an LVA-associated element, a result consistent with previous studies (Vasquez-Rifo et al., 2019).

We investigated the distribution of genes not previously linked to virulence toward *C. elegans* between high-risk and sporadic clones. For the gene *cdsA* (PA14_17120, phosphatidate cytidylyltransferase), 58.33% (7/12) of high-risk clones carried this gene, compared to 85.19% (23/27) of sporadic clones. For the gene *clpP*, none of the high-risk clones carried the corresponding subelement, while 14.81% (4/27) of the sporadic clones did. Regarding the *wecA* gene, the subelement containing this gene was absent in high-risk clones but present in 25.93% (7/27) of sporadic clones.

For LVA genes, such as PA14_60130, PA14_59660, PA14_59670, PA14_59680, PA14_59690, and PA14_59860, all were more frequently found in high-risk clones than in sporadic ones. A similar pattern was observed for bin5_se00011, bin19_se00022 and bin19_se00012, which are subelements associated with phages.

### Biofilm-forming ability of *P. aeruginosa*

**Figure 11:**
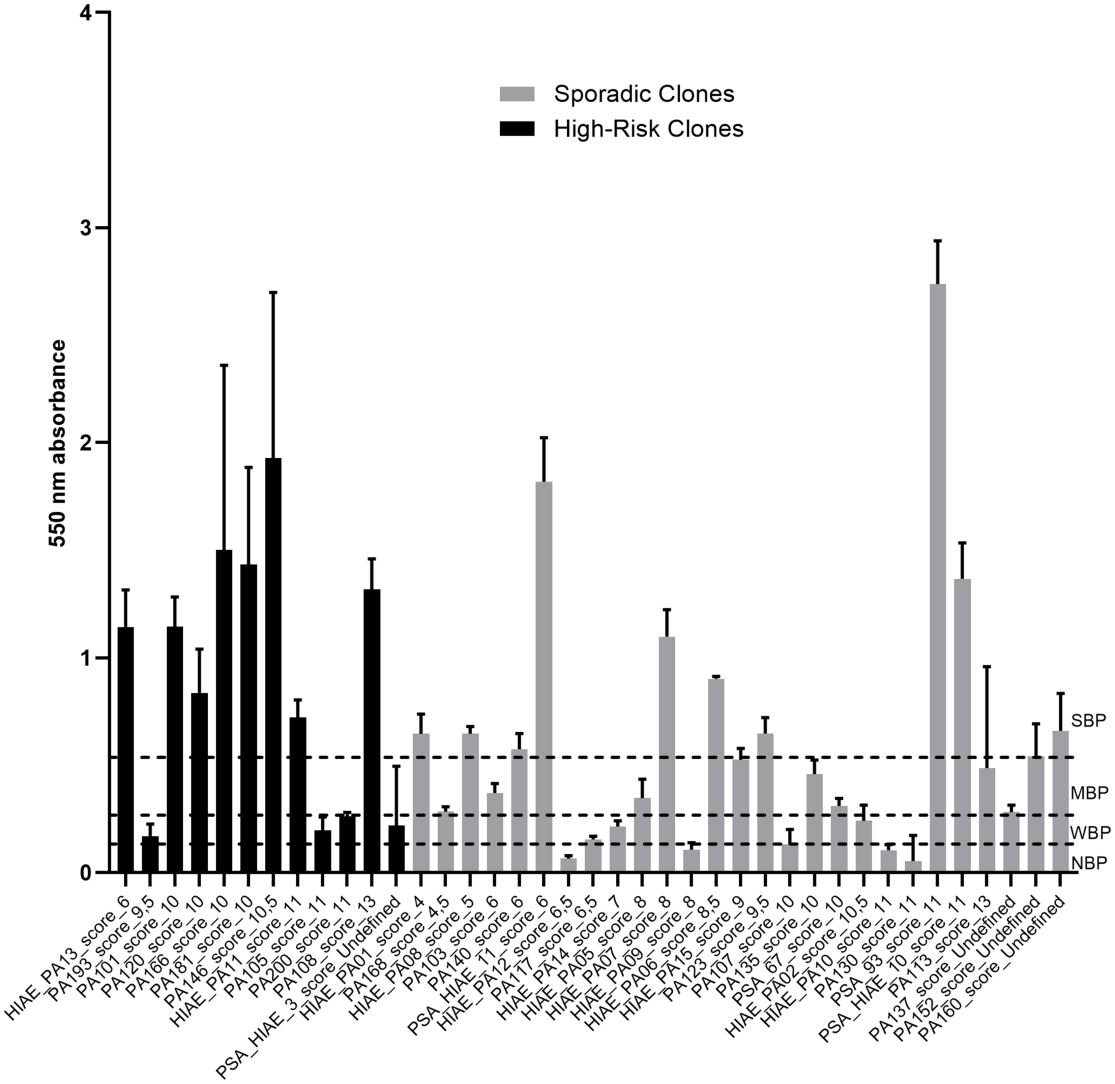
Biofilm formation by *P. aeruginosa* strains evaluated in the slow killing assay.

The ability of different isolates to form biofilm was evaluated over 24 h for the 39 strains that were subjected to the slow killing assay (Figure 11). When biofilm production was categorized into the groups NBP, WBP, MBP, and SBP, we observed that 12.82% of the strains were NBP, 15.38% were WBP, 23.08% were MBP, and 48.72% of the strains were SBP. When analyzed by clone group (high-risk clones versus sporadic clones) we observed that high-risk clones were more prolific biofilm producers than sporadic clones. High-risk clones were more represented in the WBP group (25% vs. 11.11%) and especially in the group of SBP (66.67% vs. 40.74%), and none of the strains belonging to high-risk clones were classified as NBP. In contrast, 18.52% of sporadic clone strains were classified as NBP and they were also more represented in the MBP group (29.63% vs. 8.33%). These results suggest that high-risk clones are more frequently classified as strong biofilm producers.

### Investigating HVA-associated genes and LVA-associated genes in sporadic and high-risk clones from public databases

The pangenome analysis performed with Roary on 4,835 *P. aeruginosa* genomes (3,176 sporadic clones and 1,659 high-risk clones) produced a pangenome containing 58,754 genes. The core genome was defined as consisting of 4,496 genes, with core genome genes classified as those present in ≥95% of the genomes.

To identify genes in the accessory genome associated with high-risk clones versus sporadic clones we used Scoary, collapsing linked genes that displayed identical presence/absence patterns across all sequenced isolates (e.g., organized in operons, linked to MGEs, etc.) into units and compared unit frequencies between high-risk and sporadic clones. A total of 5,966 genes were associated with high-risk genomes (*p* < 0.05, Bonferroni). However, no genes were found to be exclusively found in high-risk clones.

Among the 5,966 genes of the accessory genome, which were more frequently associated with high-risk clones, we searched for the 113 subelements found to be associated with *P. aeruginosa* virulence, either positively or negatively. For the 71 negatively associated subelements (LVA) in our 39 strains subjected to the *C. elegans* slow killing assay, 42.25% (30/71) of these subelements were found among the 5,966 genes of the accessory genome (*p* < 0.05, Bonferroni), representing a total of 37 genes. These 37 genes were mostly associated with high-risk clones, except for group_24287.

For the 42 subelements positively associated with virulence, 28.57% (12/42) were mostly found among the 5,966 genes of the accessory genome (*p* < 0.05, Bonferroni), representing a total of 23 genes. However, all of them had been mostly associated with sporadic clones.

## Discussion

In this study, we characterized the genetic and virulence factors contributing to the success of high-risk *P. aeruginosa* clones by integrating comparative genomics with an experimental infection model using *C. elegans*. Our findings reveal key insights into the epidemiological behavior, resistance mechanisms, and virulence potential of *P. aeruginosa* strains, especially those considered high-risk clones. *P. aeruginosa* is a critical public health concern due to its association with healthcare-associated infections and its increasing resistance to carbapenems (Tacconelli et al., 2018). *P. aeruginosa* is a critical public health concern due to its association with healthcare-associated infections and its increasing resistance to carbapenems (Tacconelli et al., 2018). In our study, carbapenem resistance was observed in 58.95% of isolates, which is significantly higher than national Brazilian reports in 2024 (44.17%) when considering only primary bloodstream infection and urine infection detected in ICU patients (Anvisa, 2024).

Although high-risk and sporadic clones carried a similar overall number of AMR genes, gene- and class-level patterns revealed a distinctive high-risk resistome signature. Of the 67 genes/variants surveyed, 15 (22.38%), including *aadA2*/*aadA6*, *aac(3)-Id*, *aac(6′)-Il*, *aph(6)-Id* (aminoglycosides), *dfrB5* (trimethoprim), *sul1* (sulfonamides), *cmlA6*/*floR2* (phenicols), *crpP* (fluoroquinolones), and *bla*_VIM-2_, *bla*_OXA-4_, *bla*_OXA-488_, and *bla*_PDC-35_, were significantly more frequent in high-risk clones, yielding class-level enrichment for aminoglycosides, phenicols, fluoroquinolones, trimethoprim, sulfonamides, and tetracyclines, but not for *β*-lactams or fosfomycin. Practically, this resistome signature provides surveillance flags (early recognition of high-risk clones spread via WGS screening), and highlights the importance of treatment-informing actions such as phenotypic confirmation and avoidance of drug classes signaled by these markers, with mechanism-directed options. We did not map genomic positions of AMR genes. Nevertheless, many determinants enriched in high-risk clones are well documented in the literature as mobile-element linked (e.g., *aadA2*, *aadA6*, *dfrB5*, *sul1*, *bla*_VIM-2_, *crpP*, *cmlA6*, floR2), consistent with a mobility-linked resistome signature.

Our slow-killing assay using the *C. elegans* model demonstrated substantial virulence variability across 39 isolates. *C. elegans* is a powerful discovery model for *P. aeruginosa* pathogenesis: the slow-killing assay robustly differentiates strain virulence, has revealed numerous virulence factors, and is genetically tractable and high-throughput for linking genotype to phenotype (Tan et al., 1999). Core host defenses mapped in the worm (e.g., PMK-1/p38 MAPK, SKN-1/Nrf, DAF-2/DAF-16) are conserved innate pathways relevant to infection biology, and the assay has repeatedly uncovered factors later validated in other hosts (Tan et al., 1999). At the same time, limitations must be explicit: *C. elegans* lacks an adaptive immune system and differs in aspects of innate immunity compared with vertebrates; it is maintained at 15–25 °C (not 37 °C), and its intestinal, epidermal, and respiratory analogs do not fully mirror mammalian tissues (Tran and Luallen, 2024). Consequently, some determinants essential in mammals (e.g., certain T3SS-dependent processes) are not required in the worm, and pharmacologic or tissue-specific dynamics may differ. Thus, findings from the worm are best viewed as hypothesis-generating signals that should be corroborated in mammalian models and clinical surveillance data. Virulence did not systematically align with MLST-defined high-risk clones in the *C. elegans* slow-killing model. This agrees with prior work showing an inverse trend between extensive drug resistance and nematode virulence, with clone-specific behavior: for example, ST235 and ST111 tended to be more virulent, whereas ST175 was notably hypovirulent, partly linked to an AmpR G154R allele, underscoring that high-risk status (a marker of spread and resistance) is not a proxy for virulence in this assay (Sánchez-Diener et al., 2017). Mechanistically, virulence variation is strongly shaped by the accessory genome (e.g., gain/loss of elements such as *exoU* or mobile genetic modules), which does not segregate cleanly by core-genome/MLST lineage, and genome-wide association in diverse panels confirms that accessory genes can either increase or decrease virulence toward *C. elegans* (Vasquez-Rifo et al., 2019). Clinically, high-risk clone designations (e.g., ST235, ST111, ST175) primarily reflect epidemiology of resistance and dissemination, not uniform hypervirulence, although specific determinants like *exoU* do associate with worse outcomes in mammals (Del Barrio-Tofiño et al., 2020). However, despite the known association of *exoU* with high virulence (Treepong et al., 2018), its presence did not consistently predict high virulence in our *C. elegans* model, as also shown in previous studies (Wareham et al., 2005; Feinbaum et al., 2012). Together, these data support integrating accessory-genome markers and effector genotypes, (e.g., repertoire of virulence effector genes and their key variants) with MLST when interpreting risk, and they explain why our nematode virulence readouts diverge from high-risk labels for certain clones.

Notably, we observed that LVA-associated genes/motifs were relatively enriched in MLST-defined high-risk clones, a finding that aligns with how high-risk lineages being epidemiologically defined by global dissemination and multidrug resistance, rather than by uniformly heightened virulence in model hosts. High-risk clones such as ST235, ST111, and ST175 dominate hospital outbreaks because they efficiently acquire/maintain resistance determinants via a highly plastic accessory genome (integrons, ICEs, transposons, prophages) (Kung et al., 2010), whereas their acute virulence can be clone-specific and even attenuated in *C. elegans* (e.g., ST175’s reduced nematode virulence linked to an AmpR G154R variant) (Sánchez-Diener et al., 2017). As a matter of fact, enrichment of ICE/conjugation/integrase motifs in the LVA group fits traits that promote persistence and spread under antibiotic selection rather than acute virulence in the nematode model: we found 10 ICE-related hits (e.g., integrating conjugative element protein/membrane protein), 9 conjugative transfer/shufflon (e.g., TraD coupling protein), 5 integrase/recombinase hits, and 2 phage/terminase hits (Supplementary Table S5). ICEs and related MGEs are central to AMR and adaptive traits in *P. aeruginosa*, while Pf-family prophages modulate biofilm architecture and can alter virulence/transmission features (terminase/phage hits) (Secor et al., 2020). These attributes would be advantageous in hospital niches and under therapy pressure but do not necessarily increase killing of *C. elegans*, possibly explaining why they were mostly associated with the LVA group. These observations reinforce that MLST/high-risk labels capture dissemination and resistance, whereas virulence potential is largely encoded in the accessory genome and effector genotype, arguing for combined use of WGS-based high-risk assignment combined with accessory-genome and effector profiling in surveillance and risk interpretation (Sánchez-Diener et al., 2017).

Interestingly, a considerable proportion (42.25%) of low-virulence-associated (LVA) subelements overlapped with genes enriched in high-risk clones, indicating that reduced virulence in *C. elegans* may, paradoxically, be linked to genomic features characteristic of clinically successful lineages. In contrast, high-virulence-associated (HVA) subelements were more frequently identified among sporadic clones, suggesting that virulence, as measured in the *C. elegans* model, may not be a primary determinant of clonal expansion in high-risk lineages. This discrepancy reinforces the idea that factors beyond virulence, such as antimicrobial resistance, transmission potential, and persistence mechanisms, may drive the global dissemination of high-risk clones.

In our cohort, high-risk clones were strong biofilm producers (66.7% vs. 40.7%), and none were non-producers, consistent with prior observations that high-risk lineages favor persistence phenotypes (Mulet et al., 2013). Biofilm capacity is a key strategy for environmental colonization, antibiotic tolerance, and recurrent/persistent infection, contributing to the clonal success of high-risk *P. aeruginosa* (Mulet et al., 2013; Papa-Ezdra et al., 2024). In healthcare, biofilms dominate device-associated infections (urinary catheters, orthopedic hardware, intravascular catheters), where biofilm-mediated diffusion barriers reduce antimicrobial penetration and compromise responses to treatment (Mulet et al., 2013; Lima et al., 2017). Operationally, this means isolates that combine high-risk status and strong biofilm phenotype should be flagged for enhanced prevention. At the surveillance level, coupling WGS high risk-clone tracking with a biofilm alert helps target environmental checks and earlier intervention. Managing biofilm-associated infections is therefore central to reducing healthcare burden from high-risk *P. aeruginosa* (Silva et al., 2023).

Beyond canonical systems, our GWAS also nominated *clpP* (caseinolytic protease) and *cdsA* (CDP-diacylglycerol synthase). In *P. aeruginosa*, ClpP1/ClpP2 contributes to alginate regulation and biofilm development, and participates in activation of the pyoverdine *σ*-factor cascade (PvdS) in a cell-surface signaling pathway, mechanisms that plausibly enhance persistence and host interaction (Bishop et al., 2017; Mawla et al., 2021). By contrast, *cdsA* encodes a central step in phospholipid biosynthesis; while *P. aeruginosa* adjusts virulence and stress programs to membrane-lipid remodeling, and *cdsA* perturbations are known to alter membrane physiology and antibiotic response in other bacteria, a direct virulence role for *P. aeruginosa cdsA* has not been established (Sutterlin et al., 2014; Caliskan et al., 2023). Taken together, our data newly implicate *clpP* and *cdsA* as contributors to pathogenesis in *C. elegans*, likely via biofilm or stress resilience and membrane remodeling, and they motivate targeted validation.

Noteworthy, our dataset and study design were optimized for genomic/phenotypic discovery, not for clinical outcome inference. Exploratory, post-hoc analyses relating virulence and resistance features to 30-day mortality did not reveal robust associations (data not shown). The analyses were underpowered for outcome inference and confounded by limited covariate control, so we did not interpret them further.

In summary, using a genomically diverse strain panel, bacterial GWAS can reveal previously unrecognized accessory-genome elements (AGEs) that shape virulence. Our data contributes to previously published results highlighting the contribution of specific AGEs in virulence modulation toward *C. elegans* (Vasquez-Rifo et al., 2019). In parallel, comparative genomics that links virulence phenotypes with WGS recovers accessory virulence *loci* at scale, underlining population-level genomics as a powerful discovery engine for this species that shows such great genomic diversity (Allen et al., 2020). As a matter of fact, within our HVA set, enrichment of pyoverdine-system genes (*fpvA/pvdE/pvdD*) is clinically informative because under iron limitation *P. aeruginosa* deploys pyoverdine to capture iron and, via FpvA/FpvR/PvdS, up-regulates exotoxin A and *PrpL* (Beare et al., 2003). Detection of *fpvA/pvd* modules can flag isolates for enhanced infection-control attention in ICUs and burn/respiratory units where iron scarce infection sites are the norm. Also of clinical relevance, this same iron dependence is exploited by, for example, cefiderocol, a siderophore cephalosporin which uses iron-uptake pathways to enter cells and is a key option against difficult-to-treat *P. aeruginosa* (Tamma et al., 2024). Since both cefiderocol and pyoverdine compete for the same extracellular iron, a bacterium can develop resistance by changing its pyoverdine production. In this work we observed high-risk clones consistently expressing higher levels of pyoverdine. Finally, by highlighting targets in iron uptake (pyoverdine), LPS/lipid-A pathways, and regulatory/biofilm modules (e.g., ClpP), our results motivate anti-virulence strategies that attenuate disease while imposing less selective pressure for resistance than bactericidal regimens (Liao et al., 2022), and detection of phage/terminase signatures flags mobile-element dynamics that modulate biofilm and virulence and deserve monitoring during hospital spread (Nkemngong and Teska, 2024).

## Data Availability

The datasets presented in this study can be found in online repositories. The names of the repository/repositories and accession number(s) can be found in the article/Supplementary material.

## Footnotes

^1^ https://cgc.umn.edu

^2^ https://github.com/tseemann/prokka

^3^ https://github.com/tseemann/abricate/

^4^ https://www.pseudomonas.com/

^5^ https://github.com/ncbi/amr

^6^ https://github.com/Sandramses/PAst

^7^ https://pubmlst.org/bigsdb?db=pubmlst_paeruginosa_isolates

^8^ https://www.ncbi.nlm.nih.gov/pathogens/isolates/

^9^ https://github.com/Ecogenomics/CheckM

^10^ https://github.com/tseemann/prokka

^11^ https://sanger-pathogens.github.io/Roary/

^12^ https://github.com/AdmiralenOla/Scoary

^13^ https://github.com/tseemann/snippy

^14^ http://www.iqtree.org/

^15^ https://github.com/isana18/PaeGWAS

^16^ https://www.ebi.ac.uk/interpro/
